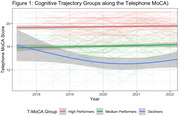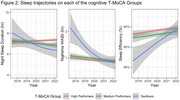# Trajectories of cognitive function and longitudinal trends in actigraphy derived measure of sleep health in older adults:Einstein Aging Study

**DOI:** 10.1002/alz70857_106939

**Published:** 2025-12-25

**Authors:** Angel Garcia De La Garza, Carol A. Derby, Qi Gao, Laura A. Rabin, Orfeu M. Buxton, Lindsay Master, Mindy J. Katz, Richard B. Lipton, Cuiling Wang

**Affiliations:** ^1^ Albert Einstein College of Medicine, Bronx, NY, USA; ^2^ Department of Neurology, and Department of Epidemiology and Population Health, Albert Einstein College of Medicine, Bronx, NY, USA; ^3^ Brooklyn College of the City University of New York, Brooklyn, NY, USA; ^4^ The Graduate Center, CUNY, New York, NY, USA; ^5^ The Pennsylvania State University, University Park, PA, USA

## Abstract

**Background:**

Sleep problems are common in older adults and have been linked with risk for cognitive impairment. Data regarding associations between longitudinal changes in sleep and changes in cognitive performance among older adults are limited. Our goal was to examine whether trajectories of actigraphically defined sleep parameters differed for individuals with different patterns of change in global cognition.

**Methods:**

Analyses included 219 Einstein Aging Study participants (mean age = 77.50, SD = 5.01; 69.86% female; 47.94% Non‐Hispanic White, 42.01% Non‐Hispanic, 10.05% Hispanic; 23.74% MCI; median follow‐up = 4 years, dementia‐free). Participants wore an actigraphy watch 24 hours/day for 16 days annually (2017–2022). Standard algorithms extracted sleep duration, wake after sleep onset (WASO), sleep efficiency, sleep midpoint, and napping. Cognition was assessed via the validated 22‐item telephone Montreal Cognitive Assessment (T‐MoCA; normal cognition > 18). Latent class mixed‐effects models identified cognitive trajectories, accounting for learning effects. Generalized additive mixed‐effects models characterized sleep patterns across cognitive groups, adjusting for age, gender, and race/ethnicity.

**Results:**

We identified three T‐MoCA cognitive trajectory groups: (1) Consistently High‐performance (*N* = 126) scoring across follow‐up (mean above 18), (2) Medium‐performance (*N* = 82) over time (mean score 15.7), and (3) Declining performance (*N* = 11) with an initial mean score of 15.2. These cognitive trajectory groups exhibited distinct longitudinal patterns of night‐time sleep duration (*p* = 0.001), WASO (*p* < 0.001), and sleep efficiency (*p* < 0.001). Night‐time sleep duration started higher and decreased more steeply in the decliner group, decreased more gradually in the medium group, and remained consistent in the high T‐MoCA group. WASO and efficiency appeared to improve over time, with the greatest improvement in Low‐performers and only gradual change in the medium and high groups. We found no significant interactions for sleep midpoint, and duration of napping.

**Conclusions:**

Actigraphy‐based sleep changes over five years differ by cognitive trajectories. Larger sleep changes in the declining group in the group with declining T‐MoCA scores may suggest that underying brain changes in those with more rapid cognitive decline impact sleep, although this should be confirmed in a larger sample.